# Dexamethasone Regulates EphA5, a Potential Inhibitory Factor with Osteogenic Capability of Human Bone Marrow Stromal Cells

**DOI:** 10.1155/2016/1301608

**Published:** 2016-01-10

**Authors:** Tsuyoshi Yamada, Toshitaka Yoshii, Hiroaki Yasuda, Atsushi Okawa, Shinichi Sotome

**Affiliations:** ^1^Department of Orthopaedic and Spinal Surgery, Graduate School, Tokyo Medical and Dental University, Tokyo 113-8510, Japan; ^2^Global Center of Excellence (GCOE) Program, International Research Center for Molecular Science in Tooth and Bone Diseases, Tokyo Medical and Dental University, Tokyo 113-8510, Japan; ^3^Section of Regenerative Therapeutics for Spine and Spinal Cord, Tokyo Medical and Dental University, Tokyo 113-8510, Japan

## Abstract

We previously demonstrated the importance of quality management procedures for the handling of human bone marrow stromal cells (hBMSCs) and provided evidence for the existence of osteogenic inhibitor molecules in BMSCs. One candidate inhibitor is the ephrin type-A receptor 5 (EphA5), which is expressed in hBMSCs and upregulated during long-term culture. In this study, forced expression of EphA5 diminished the expression of osteoblast phenotypic markers. Downregulation of endogenous EphA5 by dexamethasone treatment promoted osteoblast marker expression. EphA5 could be involved in the normal growth regulation of BMSCs and could be a potential marker for replicative senescence. Although Eph forward signaling stimulated by ephrin-B-Fc promoted the expression of ALP mRNA in BMSCs, exogenous addition of EphA5-Fc did not affect the ALP level. The mechanism underlying the silencing of EphA5 in early cultures remains unclear. EphA5 promoter was barely methylated in hBMSCs while histone deacetylation could partially suppress EphA5 expression in early-passage cultures. In repeatedly passaged cultures, the upregulation of EphA5 independent of methylation could competitively inhibit osteogenic signal transduction pathways such as EphB forward signaling. Elucidation of the potential inhibitory function of EphA5 in hBMSCs may provide an alternative approach for lineage differentiation in cell therapy strategies and regenerative medicine.

## 1. Introduction

Human bone marrow stromal cells (hBMSCs) are an attractive source for bone tissue engineering applications because of their proliferative capacity and multipotency [1, 2]. However, their differentiation potential deteriorates over multiple cell divisions [3–5], and it may thus be difficult to obtain a sufficient number of effective cells for clinical applications through ex vivo expansion. Thus, the clinical application of BMSCs requires a more complete understanding of the mechanisms that lead to the senescence of these cells.

Our previous study revealed that long-term passaged BMSCs are capable of forming bone but can also inhibit bone formation. In particular, we demonstrated the importance of quality management procedures for the handling of hBMSCs and provided evidence for the existence of osteogenic inhibitor molecules in BMSCs. One candidate inhibitor is the ephrin type-A receptor 5 (EphA5), which is expressed at low levels at early passages of hBMSC primary culture and upregulated during long-term culture [5]. EphA5 is a member of the ephrin receptor tyrosine kinase subfamily and can bind ephrins A1, A2, A3, A4, and A5. The ephrin receptors are divided into 2 groups based on the similarity of their extracellular domain sequences and their affinities for binding ephrin-A and ephrin-B ligands. The ephrin receptors have the ability to induce both forward and reverse (bidirectional) signaling between adjacent interacting cells. Recently, ephrins and their receptors were reported to be involved in bone metabolism. Zhao et al. demonstrated that signaling between the extracellular domains of ephrin-B2 expressed on osteoclasts and EphB4 in osteoblasts suppresses osteoclast differentiation and stimulates osteogenic differentiation [6]. In addition to osteoclast-osteoblast interactions, osteoblast–osteoblast interactions through ephrin A2 and either EphA2 or EphA4 have also been shown to occur [7, 8]. We found that downregulation of endogenous EphA5 using specific siRNAs or dexamethasone (DEX) treatment promoted osteoblast marker expression, suggesting that EphA5 is a potential inhibitor of bone formation [5]. However, there have been no reports on the role of EphA5 in bone metabolism, and the mechanism underlying the inhibitory effect of EphA5 on the osteogenic differentiation of BMSCs remains unclear.

BMSCs are heterogeneous and contain subpopulations of osteoprogenitors and undifferentiated cells, and it is difficult to assess the overall expression profile of each batch of cells in a clinical setting [9]. DEX, which has been used to differentiate BMSCs into adipogenic [1], chondrogenic [10–12], and osteogenic lineages [10, 13], affects not only the proliferation rate but also the subpopulation composition of BMSCs. However, the precise mechanism of how DEX induces differentiation is still unclear. Previously, we hypothesized that DEX does not directly induce BMSCs into specific lineages but rather augments the responsiveness of BMSCs to other differentiation reagents applied together with DEX. We reported that DEX induced selective proliferation of cells with higher differentiation capability not only during the initial proliferation culture but also during subsequent osteogenic induction [14] and that cells that had higher responsiveness to BMP stimulation selectively proliferated under continuous DEX treatment [15]. Moreover, DEX treatment selectively suppressed EphA5 expression [5]. Therefore, EphA5 may be a potential negative prognostic indicator of the responsiveness of BMSCs to differentiation reagents, and it may be involved in the senescence or reduced differentiation potency of BMSCs.

The goals of our research were to determine the effects of EphA5 on BMSC quality and to clarify the inhibitory mechanism involved in the reduction of differentiation potential after repeated cell division. Here, we demonstrated the inhibitory effects of EphA5 on osteogenic differentiation in hBMSCs and investigated its function and mechanism of action, together with the association between EphA5 and DEX treatment. Our findings suggest that EphA5 may be a new therapeutic target and quality control marker for the osteogenic differentiation capability of hBMSCs.

## 2. Material and Method

All of the experiments in this study were specifically approved by the Review Board of the Tokyo Medical and Dental University and were performed in accordance with the Declaration of Helsinki and university guidelines for the care and use of human subjects. Participants provided their written informed consent to participate in this study.

### 2.1. Primary Culture of hBMSCs

After informed consent was obtained, BMSCs were cultured from bone marrow aspirates of 34 patients who had received hip surgery at Tokyo Medical and Dental University under a protocol that was approved by the institutional review board. The donors ranged in age from 30 to 87 years. Approximately 2 mL of bone marrow aspirate was obtained from the medullary cavity of the femoral shaft of each patient using a bone marrow biopsy needle (Cardinal Health, Dublin, OH, USA). The aspirate was added to 20 mL of growth medium [Dulbecco's modified Eagle medium (DMEM), Sigma-Aldrich Co., St. Louis, MO, USA] containing 10% fetal bovine serum (Life Technologies Co., Carlsbad, CA, USA) and 1% antibiotic-antimycotic (10,000 U/mL penicillin G sodium, 10,000 *μ*g/mL streptomycin sulfate, and 25 *μ*g/mL amphotericin B; Life Technologies) that contained 200 IU of sodium heparin (Mochida Pharmaceutical Co. Ltd., Tokyo, Japan) and then centrifuged to remove the fat layer. Bone marrow cells were then resuspended in growth medium, and aliquots of the cell suspensions were used to count nucleated cells after hemolysis. Subsequently, 1 × 10^8^ nucleated bone marrow cells were plated into two 225 cm^2^ flasks (Becton, Dickinson and Company, Franklin Lakes, NJ, USA). The cells were then cultured in each medium at 37°C in a humidified atmosphere containing 95% air and 5% CO_2_, and the medium was replaced every three days. When primary cultures became nearly confluent, the cells were detached with 0.25% trypsin containing 1 mM EDTA (Life Technologies) and subsequently replated for each assay. Cells were passaged at a density of 2 × 10^3^ cells/cm^2^, and hBMSCs at passages 1 (P1), P5, and P10 were stored in liquid nitrogen until further use. The collected hBMSCs were either cultured or preserved separately; the cells from individual donors were assayed independently to prevent cross contamination of hBMSCs from different donors.

### 2.2. Osteogenic Differentiation

BMSCs were replated at 2 × 10^3^ cells/cm^2^ in a six-well culture plate. When the culture plates became 80% confluent, the culture media of each group were changed to osteogenic media containing 10 mM *β*-glycerophosphate (*β*-GP, Sigma-Aldrich Co.) and 50 *μ*g/mL ascorbic acid phosphate (AA, Wako, Osaka, Japan) [13] with or without 100 nM dexamethasone (DEX, Life Technologies) and/or BMP-2. After zero and seven days of osteogenic culture, the cells were used in each assay.

### 2.3. RNA Isolation, Real-Time RT-PCR

Total RNA was isolated from the culture dishes using RNeasy Mini Kits (Qiagen, GmbH, Hilden, Germany), and first-strand cDNA was prepared using the PrimeScript RT Reagent Kit with gDNA Eraser (Takara Bio Inc., Shiga, Japan) according to the manufacturer's instructions. Gene expression was quantified by real-time polymerase chain reaction (PCR) using the Mx3000P QPCR System (Agilent Technologies, Inc., Santa Clara, CA, USA) and GoTaq qPCR Master Mix (Promega Co., Fitchburg, WI, USA). Primer sets were predesigned and purchased from Takara Bio Inc. (Table 1). Standards were generated from one specific sample, and every PCR reaction in this study was run with a standard curve to quantify the relative Ct values of the samples. *β*-actin was used to normalize the amount of template that was present in each sample.

### 2.4. Lentivirus Production and Transduction

Lentivirus production was performed using the GeneCopoeia HIV-Based Lentiviral Expression System (GeneCopoeia, Inc., MD, USA): 1.3 × 10^6^ GeneCopoeia 293Ta lentiviral packaging cells were plated in a 10 cm cell culture dish (Greiner Bio-One, Frickenhausen, Germany, Cat. number 664-960) in 10 mL of DMEM supplemented with 10% heat-inactivated fetal bovine serum and incubated at 37°C with 5% CO_2_ for 24 hours before transfection.

**Figure 1:**
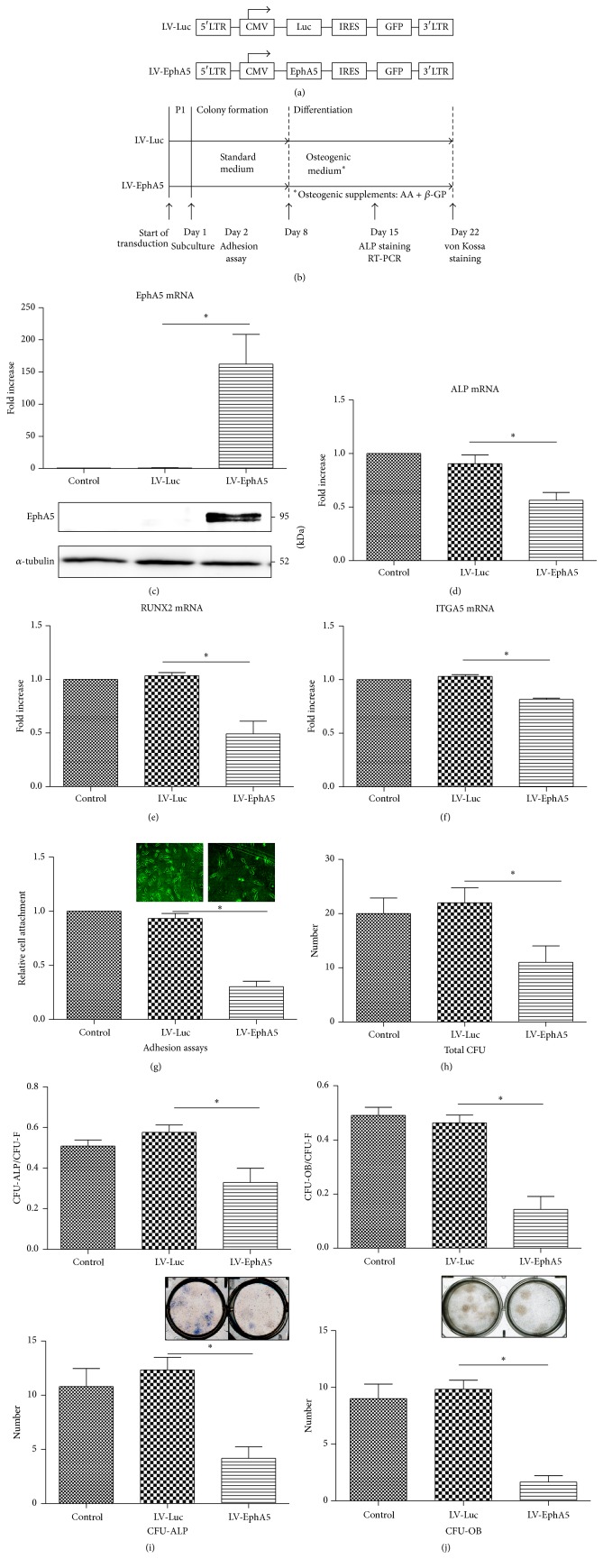
Forced expression of EphA5 inhibits osteoblast differentiation in hBMSCs. (a) Lentiviral constructs for transducing Luc and EphA5. (b) Schematic representation of the cell culture protocol. Adult hBMSCs were transduced with LV-Luc or LV-EphA5. ((c)–(f)) Quantitative analysis of mRNA expression of osteogenic markers at 7 days of osteogenic culture: (c) EphA5, (d) ALP, (e) Runx-2, and (f) ITGA5 (*n* = 4). The fold change of gene expression was normalized against the expression in cell cultures without LV transduction. ((c), lower) EphA5 protein levels were determined by western blot analysis. (g) Adhesion assays (*n* = 4). Images of cell attachment (upper). ((h)–(j)) CFU assays (*n* = 4). (h) Number of total colonies. (i) CFU-ALP positive rate (upper) and number of ALP-positive colonies (lower). Images of wells after ALP staining (middle). (j) CFU-OB positive rate (upper) and number of OB-positive colonies (lower). Images of wells after von Kossa staining (middle).

In a sterile polypropylene tube, we diluted 2.5 *μ*g of lentiviral ORF expression vector plasmid (GeneCopoeia) (Figure 1(a)), 1.25 *μ*g of packaging plasmid, pCAG-HIVgp (a gift from Dr. Miyoshi, RIKEN), and 1.25 *μ*g of VSV-G, Rev plasmid, and pCMV-VSV-G-RSV-Rev (a gift from Dr. Miyoshi, RIKEN) into 200 *μ*L of Opti-MEM l (Invitrogen). In a separate tube, we diluted 15 *μ*L of FuGENE HD Transfection Reagent (Promega, WI, USA) into Opti-MEM l. We added the diluted FuGENE dropwise to the DNA solution and incubated the mixture for 15 minutes at room temperature. We added the DNA-FuGENE complex directly to each dish and incubated the cells in a CO_2_ incubator at 37°C overnight. The overnight culture medium was replaced with fresh DMEM medium supplemented with 4% heat-inactivated fetal bovine serum, penicillin-streptomycin, and 1/500 volume of TiterBoost reagent (GeneCopoeia). Supernatants were collected 48 h after transfection, filtrated through 0.45 *μ*m polyethersulfone (PES) low-protein-binding filters (Whatman, NJ, USA), and concentrated 40 times by Lenti-X Concentrator (Clontech) according to the manufacturer's protocols. The concentrated supernatant containing lentiviral particles was used directly to determine the titer and to transduce target cells in vitro. Lentiviral stocks were aliquoted and stored at −80°C. The titer of each batch of lentiviral vectors was assessed using the Lenti-X qRT-PCR titration kit (Clontech).

We plated 2 × 10^4^ of the target P1 hBMSCs per well in a 24-well plate 24 h prior to viral infection. For each well, 0.5 mL of virus suspension was diluted in growth medium with polybrene at a final concentration of 4 *μ*g/mL. We infected BMSCs by replacing the growth medium over the cells with the diluted viral supernatant [multiplicity of infection: 250 (MOI = 250)] and incubating the cells for 2 h at 4°C, followed by transfer to a 37°C incubator with 5% CO_2_ and incubation overnight. The viral supernatant medium was then removed and lentivirus- (LV-) transduced BMSCs were replated at a density of 80 cells/cm^2^ (clonal density) or 2 × 10^3^ cells/cm^2^ onto a 6-well plate and used for further assays.

### 2.5. Adhesion Assays

Culture plates for adhesion assays were coated with 7 *μ*g/mL collagen I for 1 h at 37°C and then blocked with 1% BSA in DMEM. LV-transduced BMSCs were replated at a density of 2 × 10^3^ cells/cm^2^ onto a 6-well plate precoated with collagen I incubated for 24 h at 37°C. At 2 days after transduction, the nonadherent cells were removed by extensive, aggressive washing with PBS. The remaining adherent cells were then trypsinized and counted in a hemocytometer. Nontreated cells were used as a control. To calculate relative cell attachment, the number of attached cells was normalized to the number of control attached cells.

### 2.6. CFU Assay

For clonal analysis, colony-forming unit (CFU) assays were performed. BMSCs were plated at 800 cells/well in 6-well culture plates and maintained in each medium for 7 days to form single cell-derived colonies, respectively. Then, the medium was changed to an osteogenic medium (CFU-ALP and CFU-OB).

### 2.7. CFU-ALP

Dishes were stained for ALP at 7 days of osteogenic induction. Dishes fixed with 10% neutral buffered formalin were washed with PBS and then incubated with a filtered mixture of naphthol AS-MX phosphate (0.1 mg/mL, Sigma-Aldrich Co.), N,N-dimethylformamide (0.5%, Wako), MgCl_2_ (2 mM), and Fast Blue BB salt (0.6 mg/mL, Sigma-Aldrich) in 0.1 M Tris-Cl (pH 8.5) for 30 min at room temperature.

### 2.8. CFU-OB

Mineralized colonies were identified by von Kossa staining and designated as colony-forming unit-osteoblasts (CFU-OB). At 14 days of osteogenic induction, cells were washed twice with Gey's balanced salt solution, fixed with 10% formalin, rinsed with 0.1 mol/L cacodylic buffer, and covered with 1.0 mL of 5% silver nitrate (Wako). The cells were then exposed to UV light for 1 h. Finally, the dishes were rinsed with distilled water and air-dried.

After positive colonies were counted in each assay, the dishes were stained with crystal violet to visualize all colonies present on the dishes, and the total number of colonies was determined. Colonies with a diameter < 2 mm and faintly stained colonies were ignored.

### 2.9. von Kossa Staining for Calcium Deposits

Cells were fixed with 10% formalin for 10 min at 4°C and washed with water three times, after which the cells were incubated with 3% silver nitrate for 60 min and exposed to light from a 40 W lamp. After rinsing with water, the cells were incubated with 5% sodium thiosulfate for 2 min followed by washing with water.

### 2.10. Western Blot Analysis

Proteins were extracted from hBMSCs. Total cellular protein was prepared by lysing cells in RIPA buffer at various time points. The protein concentration was determined using the BCA Protein Reagent Kit (Pierce, Rockford, IL). A primary antibody for EphA5 (AP7610d, ABGENT) and *α*-tubulin (11H10) rabbit mAb (#2125S, Cell Signaling Technology, Inc., Tokyo, Japan) were obtained. Protein (15 *μ*g) was separated by 10% SDS-PAGE and then transferred to a polyvinylidene difluoride (PVDF) membrane. After blocking with PVDF Blocking Reagent for* Can Get Signal* (TOYOBO Life Science, Tokyo, Japan), membranes were hybridized with the primary antibody overnight at 4°C and then hybridized with HRP-linked anti-rabbit immunoglobulin G secondary antibody (#7074, Cell Signaling Technology, Inc., Tokyo, Japan) for 1 h at room temperature. We then added Lumigen TMA-6 (Lumigen Inc., Southfield, MI, USA) onto the membrane and let it stand for 2 minutes. The signals were detected using an enhanced chemiluminescence method on an ImageQuant LAS4000 Series system (GE Healthcare UK Ltd., England).

### 2.11. Microarray Analysis

The total RNA from subconfluent nontreated BMSCs and BMSCs treated with DEX for 6 h at P5 was isolated and then treated with DNase1 for microarray analysis. Total RNA (1 *μ*g) was amplified and labeled with Cy3 or Cy5 using the Ambion Amino Allyl MessageAmp II aRNA Amplification Kit (Life Technologies, Cat. Number 1753) according to the manufacturer's instructions. An Agilent Whole Human Genome Microarray 4 × 44 K (G4110F) was hybridized with 825 ng of amplified RNA at 65°C for 16 h and washed using a Fluidics Station 450 system (Affymetrix, Inc., Santa Clara, CA, USA). The microarray slides were scanned using a GenePix 4000B scanner from Axon Instruments (Union City, CA, USA), and each microarray image was first analyzed using GenePix Pro 6.1 image analysis software (Molecular Devices, LLC, Sunnyvale, CA, USA) to determine the Cy3 and Cy5 fluorescence intensity and background noise for all spots on the array. The microarray data were normalized to the median value, and log_2_ ratios were calculated versus the values for the nontreated groups of the corresponding donor sample (*n* = 9). Genes that were up- or downregulated by more than threefold relative to the median value for all donor samples were further classified according to their protein types using the SOSUI program (http://bp.nuap.nagoya-u.ac.jp/sosui/).

### 2.12. Flow Cytometry Analysis

BMSCs cultured on a 100 mm culture dish with or without DEX treatment (100 nM, 14 days) were harvested by trypsinization. To identify ALP-positive cells, the collected cells were incubated for 30 min at 4°C with mouse monoclonal anti-human ALP antibody (R&D systems, Inc., Minneapolis, MN, USA) and washed and incubated with FITC-conjugated secondary antibody (rat anti-mouse IgG1 monoclonal antibody; Becton, Dickinson and Company) for 30 min at 4°C. Flow cytometric analyses were performed using a FACS Calibur system.

### 2.13. Ephrin-Fc and EphA5-Fc Treatment

Subconfluent monolayers of cells in 6-well plates were incubated with 0, 0.4, or 4 *μ*g/mL Fc (6-001-A, R&D Systems, Inc., MN, USA), ephrin-Fc (SMPK3, R&D Systems, Inc.), or recombinant rat EphA5-Fc chimera (541-A5, R&D Systems, Inc.) in DMEM containing 10% fetal bovine serum, 50 *μ*g/mL ascorbic acid (Sigma-Aldrich Co.), and 10 mM *β*-glycerophosphate (Wako) for the indicated times. The mRNA expression level of ALP at day 7 was measured in P1 and P5 cells (*n* = 4).

### 2.14. 5-Azacytidine and VPA Treatment

Valproic acid (VPA, P4543, Sigma, St. Louis, MO, USA) was dissolved in H_2_O at a concentration of 1 M and added to the culture medium at a final concentration of 1 mM. BMSCs were cultured in media supplemented with 10 *μ*M 5-azacytidine (A2385, Sigma) or 5-aza-2′-deoxycytidine (A3656, Sigma) for 4 days or 1 mM valproic acid for 24 h.

### 2.15. Genomic DNA Extraction and Sodium Bisulfate DNA Modification

Genomic DNA was isolated from cells and tissues using the DNeasy Kit (Qiagen) and stored at −20°C before use. Genomic DNA (1 *μ*g) from P1 cells or P5 cells was treated with sodium bisulfite according to the manufacturer's recommendations (EZ DNA Methylation-Gold Kit, Zymo Research, Orange, CA). Enzymatically methylated DNA (Universal Methylated Human DNA Standard, Zymo Research) was used as a positive control.

### 2.16. MSRE Digestion

Each reaction mixture for the OneStep qMethyl procedure was optimized for 20 ng of input DNA according to the manufacturer's recommendations (OneStep qMethyl Kit, Zymo Research, Orange, CA). The designed primers were 5′-AGGAGGCTCGGAGAAGATGC-3′ (forward) and 5′-CATCTCCCTACCTTCGTTGCTG-3′ (reverse), which amplify a DNA locus (region) that contains two methylation-sensitive restriction enzyme (MSRE) sites. The thermocycling conditions used were 37°C for 2 h during MSRE digestion; 1 cycle of 95°C for 10 minutes; 35 cycles of 95°C for 30 seconds, 63°C for 30 seconds, and 72°C for 30 seconds; and a final extension at 72°C for 7 minutes. The methylation level for amplified loci (regions) was determined using the following equation. Percent methylation = 100 × 2^−ΔCt^, where ΔCt is the average Ct value from the test reaction minus the average Ct value from the reference reaction.

### 2.17. Statistical Analysis

The values were expressed as the arithmetic mean ± standard error of the mean (SEM) and analyzed using one-way analysis of variance (ANOVA). Then, Student's *t*-test was used for between-group comparisons. After the *p* values were corrected using the Bonferroni correction, statistical significance was determined. Statistical significance is indicated by “*∗*” in the graphs.

## 3. Results

### 3.1. Forced Expression of EphA5 Suppresses Osteogenic Differentiation in hBMSCs

To overexpress EphA5 in BMSCs and determine the role of EphA5 in osteogenic differentiation, hBMSCs were transduced with a lentiviral vector encoding EphA5 and GFP (Figures 1(a) and 1(b)). The LV-EphA5-transduced cells showed increased GFP expression and increased EphA5 mRNA levels at 7 days of osteogenic culture (Figure 1(c)). EphA5 overexpression in hBMSCs decreased ALP mRNA, Runx2 mRNA, and ITGA5 mRNA expression levels (Figures 1(d), 1(e), and 1(f)) and did not affect EphA2 and EphA4 mRNA expression levels (S1 Figure, in Supplementary Material available online at http://dx.doi.org/10.1155/2016/1301608). LV-EphA5-transduced BMSCs demonstrated decreased adhesion to 6-well plates compared with LV-Luc-transduced BMSCs or control untreated BMSCs at 1 day after seeding. There were no marked differences in the cell morphology between LV-Luc- and LV-EphA5-transduced BMCSs (Figure 1(g)).

Consistent with this effect, LV-EphA5 transduction in hBMSCs decreased the osteogenic capacity of the hBMSCs as indicated by negative ALP staining and selectively inhibited their colony formation. BMSCs transduced with the lentiviral vector at passage 1 were plated at clonal density and cultured in growth medium for 7 days through the colony formation period. Each well was then incubated in osteogenic medium for an additional 7 or 14 days. Colonies were stained and ALP-positive and OB-positive colonies were counted. The total colony number and ratio of ALP- and OB-positive colonies in the LV-EphA5-treated wells were lower than those in the LV-Luc-treated wells (Figures 1(h)–1(j)). These results demonstrate that forced expression of EphA5 is sufficient to decrease not only the expression of osteoblast markers and osteogenic capacity of primary hBMSCs but also cell attachment on culture wells.

### 3.2. EphA5 is Upregulated in Late Culture and Downregulated during Osteogenic Induction with DEX

**Figure 2:**
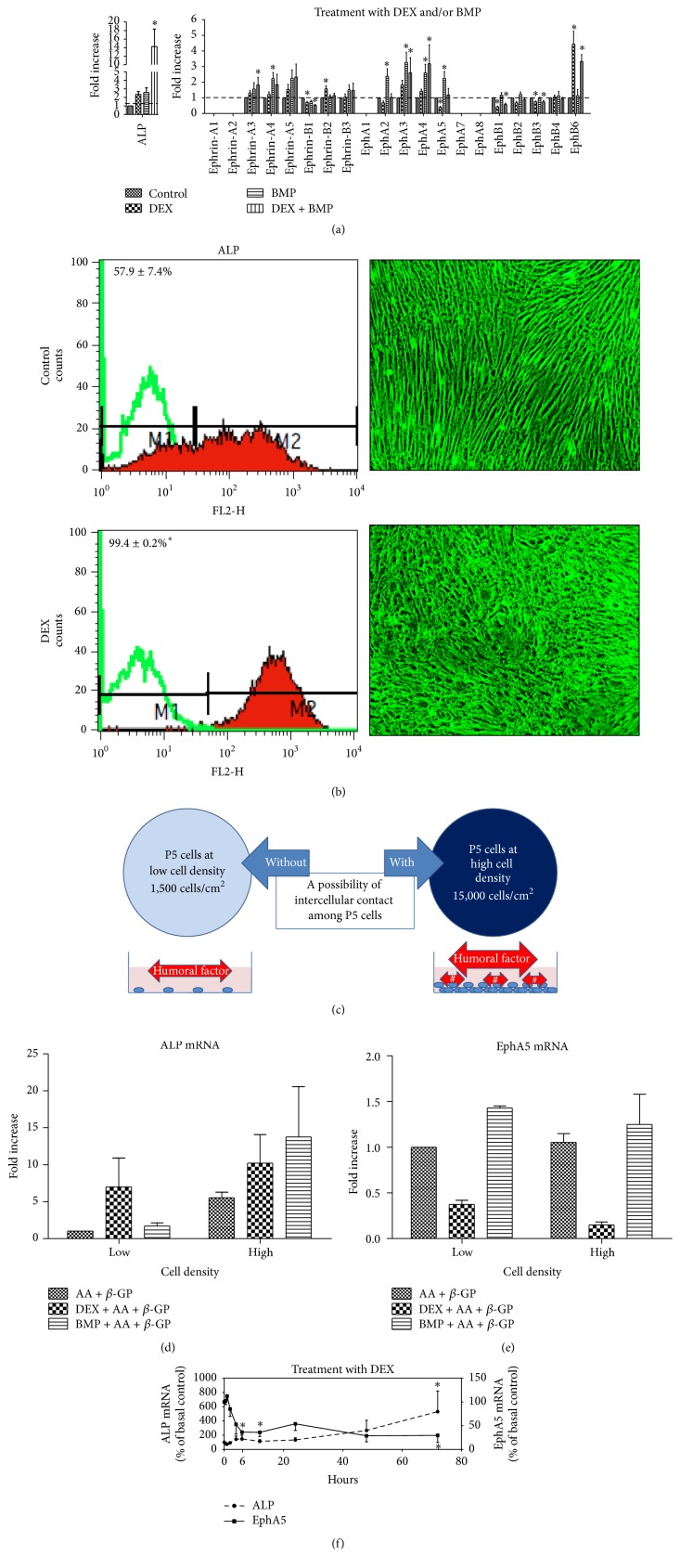
EphA5 is upregulated after repeated passaging and downregulated during osteogenic induction with DEX. (a) Expression of members of the ephrin and Eph receptor families in hBMSCs. P5 BMSCs were differentiated into osteogenic lineages using AA + *β*-GP + DEX or BMP-2, and quantitative PCR to determine the mRNA expression of ephrin and Eph receptor family members was performed at day 3 of osteogenic induction (*n* = 8). The fold change of gene expression was normalized against the expression level in the nontreated culture. DEX treatment suppressed and BMP treatment increased the mRNA expression of both EphA2 and EphA5, respectively. (b) Cell surface alkaline phosphatase (ALP) expression in hBMSCs at passage 5 was analyzed using flow cytometry (left, *n* = 4). Positive expression was defined as a level of fluorescence higher than 97% of the fluorescence obtained with the corresponding isotype-matched control antibody. Positive expression rates are displayed as the mean ± SEM (green line, isotype control). DEX-treated cells showed a higher positive rate for ALP expression than untreated cells (^*∗*^ *p* < 0.05). Cell bodies became smaller and showed morphological changes at day 14 of DEX treatment (right, magnification ×40). ((c)–(e)) Effect of cell density on ALP and EphA5 mRNA levels under different osteogenic induction protocols. (c) hBMSCs at P5 were seeded at low cell density, without intercellular contact, or at high cell density, that is, almost at confluency. # shows intracellular contact between P1 and P5 cells. ((d), (e)) Quantitative analyses of (d) ALP and (e) EphA5 mRNA expression. The fold change of gene expression was normalized against the expression in cultures at low density with AA and *β*-GP treatment (*n* = 4). (f) Quantitative analyses of ALP and EphA5 mRNA expression in BMSCs at passage 5 when treated with DEX. The change in the expression of each gene expression was normalized against the expression in cell cultures prior to DEX addition (*n* = 9). EphA5 expression was transiently reduced after 2 hours of DEX induction and reached a minimum value after 6 h of induction. ALP expression was induced after 24 h of DEX induction, after which EphA5 expression gradually decreased again.

We analyzed the expression of all known members of the ephrin and Eph families in the hBMSC cultures after osteogenic induction with DEX and BMP-2 (Figure 2(a)). When DEX treatment was continued for 72 h, the mRNA expression of ephrin-B1, EphA2, EphA5, EphB1, EphB2, and EphB3 among the ephrin receptor tyrosine kinase subfamily was decreased, whereas that of ephrin-B2 and EphB6 was increased. In contrast, BMP-2 treatment increased the expression of ephrin-A4, ephrin-A5, EphA2, EphA3, EphA4, and EphA5. The expression pattern of ephrins and Eph receptors during osteogenic induction using BMP-2 thus differed from that during osteogenic induction using DEX. A synergetic effect of DEX and BMP-2 was observed, as demonstrated by increased ALP mRNA expression. Among the ephrin and Eph families, the expression pattern of EphA5 was closely related to that of EphA2 when hBMSCs were treated with DEX and/or BMP. For example, the fall in EphA5 and EphA2 levels by DEX treatment was matched by rises in EphA3 and EphA4 levels in most samples.

We investigated the effect of continuous DEX treatment throughout the proliferation stage of BMSCs on differentiation capability by comparing DEX-treated cells with untreated cells. DEX treatment throughout the culture period led to dramatic changes in cell morphology (Figure 2(b)). Larger ALP-positive subpopulations were observed after continuous DEX treatment, which indicates that selection of osteogenic cell populations occurred (Figure 2(b)). Furthermore, we evaluated the effect of intercellular contact on the osteogenic capability of BMSCs under different induction protocols (Figure 2(c)). Augmentation of ALP mRNA expression with DEX induction was observed irrespective of cell density, whereas DEX-excluded osteogenic induction exerted a stronger effect at a high cell density than at a low cell density (Figure 2(d)), possibly because of the effect of osteogenic factors requiring cell-cell contact, such as those expressed by aligned osteoblasts, or osteogenic humoral factors released from BMSCs at high density. In contrast, EphA5 mRNA expression was minimally affected by the cell density when the cells were subjected to DEX-excluded osteogenic induction (Figure 2(e), S2 Figures A and B).

To investigate DEX-mediated osteogenesis, BMSCs at P5 were stimulated with 10^−7 ^M DEX for various durations. Although the decrease in EphA5 mRNA levels showed biphasic changes, DEX treatment inhibited EphA5 mRNA expression for up to 6 hours in most samples, followed by increased expression of ALP (Figure 2(f)). On the other hand, BMP-2 treatment showed the increase in EphA5 mRNA levels, leading to different results compared to DEX treatment (S2 Figure C).

To clarify the mechanism through which DEX promotes hBMSC osteogenesis, the differential gene expression of DEX-untreated BMSCs versus BMSCs treated with DEX for 6 h at P5 was analyzed by Affymetrix GeneChip technology for 9 independent BMSC preparations. The microarray analysis revealed that DEX treatment upregulated ITGA5 (Table 2) and its downstream target PIK3R1, in addition to FKBP5, the Src kinase family (SGEF, SHC4, SLA, and DIRAS3), FoxO1, and BMP-6. As expected, this treatment downregulated not only EphA5 but also downstream targets of EphA such as Rho/RAS-related genes (e.g., RASD1, ARHGAP, and RGNEF). Of note, DEX treatment altered the expression of various interleukins, tumor necrosis factors, and chemokine receptors (Tables 2 and 3). These dramatic changes caused by DEX induction may be a key to understanding the mechanism of osteogenic differentiation. ITGA5 upregulation induced by DEX has been reported to promote osteoblast differentiation of hBMSCs [16] and, in the present study, RT-PCR also revealed that osteogenic induction using DEX promoted ITGA5 expression at 7 days (S3 Figure). EphA5 and ITGA5 may therefore be involved in this DEX-mediated osteogenic pathway.

### 3.3. hBMSCs Treated with EphA5-Fc and Untreated hBMSCs Express ALP mRNA at Similar Levels

**Figure 3:**
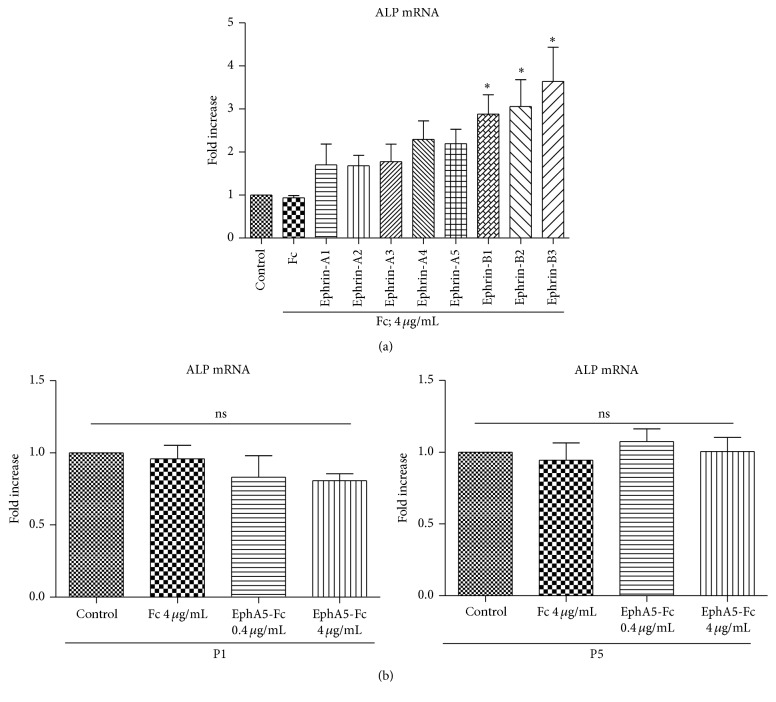
hBMSCs treated with EphA5-Fc express ALP mRNA at levels similar to those of nontreated hBMSCs. (a) hBMSCs at P5 were left untreated (control) or treated for 7 days with 4 *μ*g/mL Fc or ephrin-Fc. Quantitative analysis of ALP mRNA expression (*n* = 4). The fold change of gene expression was normalized against the expression in cell cultures without Fc treatment. (b) hBMSCs at P1 (left) or P5 (right) were left untreated (control) or treated for 7 days with 0 or 4 *μ*g/mL Fc and EphA5-Fc. Quantitative analysis of ALP mRNA expression (*n* = 4). The fold change of gene expression was normalized against the expression in cell cultures without Fc treatment. Images of wells after ALP staining (upper).

In many cell types, Eph forward signaling and ephrin reverse signaling mediate opposite effects. It is therefore important to determine which signal contributes to the deterioration of the differentiation capability of hBMSCs. To clarify the opposite effects of Eph forward signaling and ephrin reverse signaling on the differentiation capability of hBMSCs, we first used various ephrin-Fc constructs to stimulate Eph forward signaling. Treatment with ephrin-A-Fc, which mainly activates various EphA forward signaling pathways, did not affect ALP mRNA levels, while treatment with ephrin-B-Fc significantly increased ALP mRNA expression (Figure 3(a)).

We performed q-PCR assays of cells cultured in the presence of a soluble form of the EphA5 extracellular domain fused to Fc (EphA5-Fc), based on the hypothesis that the EphA5-Fc soluble receptor, upon binding to ephrin ligands, would activate ephrin reverse signaling and inhibit Eph forward signaling by competing with the endogenous EphA5 receptor to bind to the ephrin ligand in hBMSCs. However, we found that exogenous addition of soluble EphA5-Fc did not affect ALP mRNA expression levels in either P1 cells or P5 cells (Figure 3(b)).

### 3.4. Silencing of EphA5 in Early-Passage hBMSCs Is Not Associated with Aberrant Hypermethylation of Its Promoter

**Figure 4:**
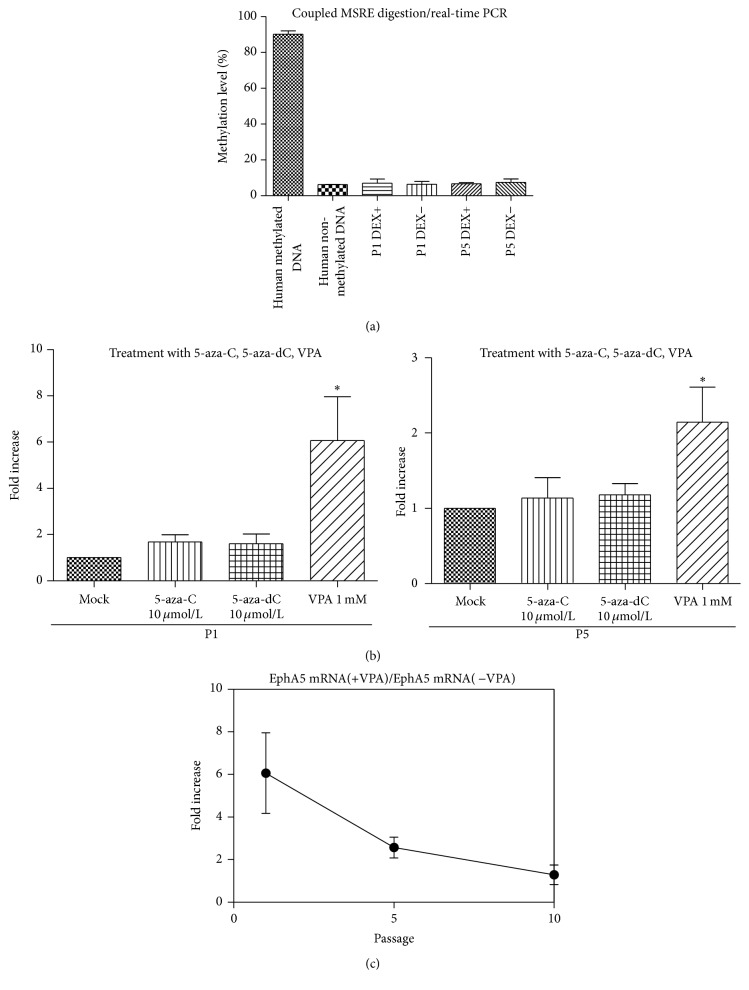
Silencing of EphA5 in early-passage hBMSCs is not associated with aberrant hypermethylation of the EphA5 promoter. (a) The methylation level for the EphA5 promoter that contains two Methylation-Sensitive Restriction Enzymes (MSRE) sites was determined (*n* = 4). The methylation level for the EphA5 promoter in hBMSCs at P1 and P5 was much lower than that in human methylated DNA used as a positive control (PC) and similar to that in human nonmethylated DNA used as a negative control (NC). (b) EphA5 mRNA expression analysis by quantitative RT-PCR in hBMSCs treated with 5-azacytidine (5-aza-C) or 5-aza-2′-deoxycytidine (5-aza-dC) for 4 days, or valproic acid (VPA) for 24 hours (*n* = 7). The fold change of gene expression was normalized to the expression in mock-treated cell cultures. (c) The fold change of in the EphA5 mRNA level caused by VPA treatment at the indicated culture passage numbers was measured (*n* = 5).

EphA5 was prominently upregulated over the course of hBMSC proliferation. This mechanism may be associated with active suppression at lower passage numbers. To further elucidate the mechanism underlying the silencing of EphA5 in early-passage P1 cells, we analyzed the 5′ regulatory region of the EphA5 gene. In particular, we analyzed a 406 bp segment of a CpG island encompassing the TSS (transcription start site) (−103 to +303 bp; TSS, +1) that contains 38 CpG dinucleotides; this segment spans the core promoter exon and part of intron 1. The methylation level for the EphA5 promoter, which contains two methylation-sensitive restriction enzyme (MSRE) sites, was determined. The methylation level for the EphA5 promoter in hBMSCs at P1 or P5 was similar to that in human nonmethylated DNA as a negative control. EphA5 promoter was barely methylated in P1 and P5 cells, even under DEX treatment (Figure 4(a)).

Treatment of hBMSCs with the DNA methylation inhibitors 5-azacytidine and 5-aza-2′-deoxycytidine did not alter EphA5 mRNA expression, indicating a lack of sensitivity of EphA5 expression to genomic DNA methylation status. However, treatment with the histone deacetylase 1 (HDAC1) inhibitor valproic acid (VPA) for 24 hours significantly increased EphA5 mRNA expression in P1 and P5 cells (Figure 4(b)). We then evaluated whether passage-dependent changes in histone deacetylation affected EphA5 expression by treating BMSCs at each passage with VPA. VPA treatment at 1 mM only slightly increased EphA expression in late-passage cells (Figure 4(c)), which suggests that histone deacetylation suppresses EphA5 expression in early-passage cultures and that chromatin remodeling through histone deacetylation is a potential mechanism for silencing of the EphA5 gene.

## 4. Discussion

Eph and Eph-related receptors have been implicated in developmental events, particularly in the nervous system [17–22]. The role of Eph receptors and ephrin ligands in cell adhesion and migration [23–25], formation of tissue compartment borders [26–28], and regulation of cell proliferation in various tumors [29–35] is also well documented; however, their potential role in bone biology is only now beginning to emerge. Among the ephrin and Eph family members expressed in hBMSCs, only EphA5 was upregulated in late-passage cultures [5]. In this study, we focused on the effects of EphA5 on BMSC osteogenic differentiation and provided data showing that EphA5 is important for regulating the osteogenic differentiation capability of hBMSCs. Gain-of-function studies showed that EphA5 diminishes the expression of osteoblast phenotypic markers. Downregulation of endogenous EphA5 by specific siRNAs or DEX treatment promoted osteoblast marker expression and osteogenic differentiation [5]. Therefore, considering that prolonged culture periods reduce the osteogenic differentiation potential of hBMSCs and EphA5 is gradually upregulated during long-term culture [5] (S2 Figure B), EphA5 could be involved in both the dormancy process and normal growth regulation of BMSCs and could be a potential candidate marker for replicative senescence. Previous reports suggested that EphA5 is involved in regulation of tumor dormancy [36, 37]. Furthermore, several studies previously indicated that cell senescence-related genes are localized on human chromosome 4, as introduction of normal human chromosome 4 into three immortal cell lines resulted in a loss of proliferation and reversal of the immortal phenotype [4, 38]. In contrast to EphA2, which resides on chromosome 1; EphA3, EphB1, EphB2, and EphB3 on chromosome 3; EphA4 on chromosome 2; and EphB4 and EphB6 on chromosome 7, EphA5 is located on chromosome 4q13, which might further support its involvement in senescence.

In many cell types, Eph forward signaling and ephrin reverse signaling mediate opposite effects [39–41]. Emerging evidence suggests that cells coexpressing Eph receptors and ephrins exist in many tissues and the coexpression of EphAs and ephrin-A results in* trans-* (between adjacent cells) or* cis-* (in the same cell) interactions [42, 43]. Coclustering occurs also between EphA and EphB receptors, resulting in activation of both receptor types that does not require the presence of both ligands, with outcomes depending on the relative receptor expression [44]. We previously found that various endogenous EphA and EphB receptors are expressed in hBMSC like tumor cells [5]. We now propose that coexpression of Eph receptors and ephrins regulates the osteogenic differentiation of hBMSCs. At early passages of hBMSC primary culture, EphA5 is expressed at low levels, independent of methylation. Under normal conditions, EphA5 and ephrin-As/ephrin-Bs are properly expressed and engaged with each other. The signaling triggered by EphA activation counteracts growth factor signaling by promoting activation of Ras/ERK and PI3K/Akt [45], which contributes to the maintenance of cell homeostasis. Repeated passaging results in selective upregulation of EphA5 but not ephrins [5] thereby leading to an excess of nonligated EphA5.

**Figure 5:**
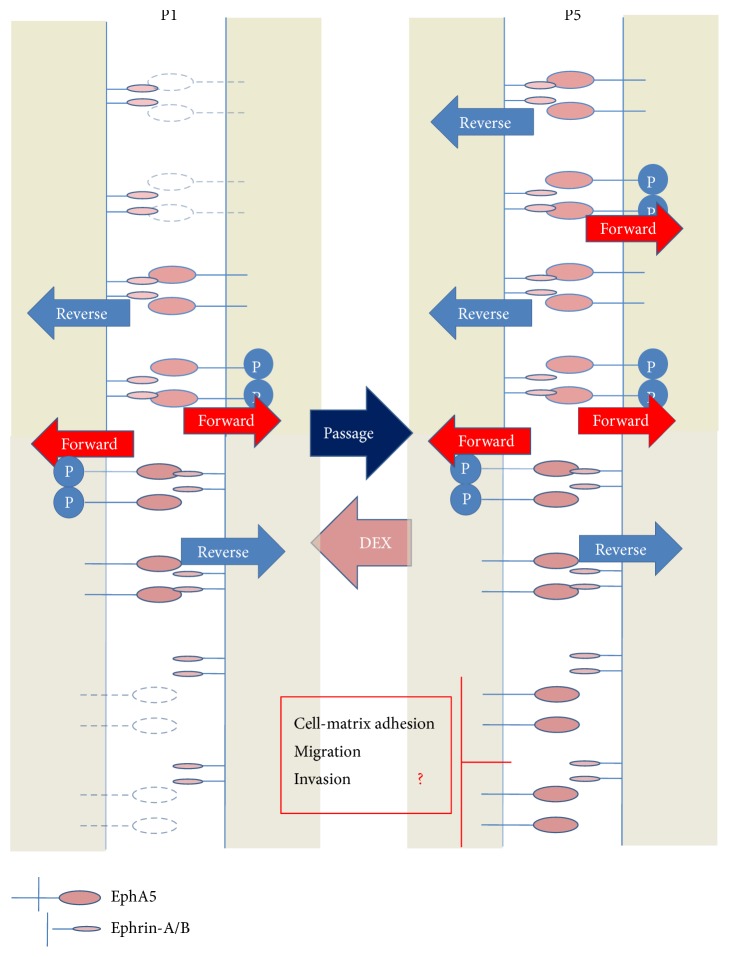
Proposed mechanisms by which EphA5 inhibits hBMSC osteogenesis. Coexpression of EphA5 and ephrins regulates hBMSC osteogenesis. Repeating passaging upregulates EphA5 but not ephrins, which results in an excess of nonligated EphA5. An imbalance between receptor and ligand expression may compromise other Eph ligand-dependent differentiation processes and promote ligand-independent suppression in hBMSCs.

In the present study, although Eph forward signaling stimulated by ephrin-B-Fc promoted the expression of ALP mRNA in BMSCs as previously reported [6, 46, 47] (Figure 3(a)), treatment with ephrin-A-Fc did not significantly affect osteogenic differentiation in vitro. In particular, exogenous addition of EphA5-Fc, which could activate ephrin reverse signaling or inhibit EphA5 forward signaling by competitive bindings, did not affect the ALP expression level. Moreover, although the expression of EphA5 at low cell density, where there is no possibility of intercellular contact among BMSCs, is nearly identical to that observed at high cell density with cell-cell contact, the ALP mRNA level is lower at low cell density. Cell-cell contact such as osteoclast-osteoblast [6], osteoblast-osteoblast [7, 8], and BMSCs-BMSCs is generally supposed to be essential for the osteogenic differentiation. In the case that there is no cell-cell contact, the essential signaling for osteogenic differentiation may not be transmitted, leading to the fall in ALP levels. It is becoming increasingly clear that at least some Eph kinases can function without ligand engagement [48]. The increased expression of EphA5 could competitively prevent various ephrins from binding to other Eph receptors and thus inhibit osteogenic signal transduction pathways such as EphB forward signaling. Besides this mechanism, there is a possibility that a large excess of nonligated EphA5 during long-term culture, under physiologically abnormal conditions, may thus have some negligible effect on maintaining the undifferentiated state of BMSCs, independently of ligand binding (Figure 5(a)). Another possibility is that the canonical EphA5 forward signaling stimulated by ephrins or the ephrin reverse signaling stimulated by EphA5 itself does not transmit osteogenic signals. Rather, EphA5 may function to attenuate the signaling of coclustered catalytically incompetent receptors such as EphA10 and EphB6, which may mediate kinase-independent forward signals similarly to kinase-inactive variant forms of other Eph receptors [49–51]. The present results are difficult to interpret because of the complexity and lack of specificity of ephrin/Eph binding; for example, each ephrin can bind to more than one Eph receptor and vice versa, even between classes A and B. Moreover, the ephrin/Eph signals are transmitted bidirectionally. In general, Fc-clustered ligands are capable of activating the receptor in vitro. Although many authors use the ephrin-Fc as the ligands which can activate Eph forward signals [6, 23, 47, 48, 52], it also remains unclear whether the ephrin-Fc actually induced receptor activation; these ligands often act as inhibitors or blocking reagents rather than activators if not added after cross-linking. The downstream signaling of EphA5 is not supposed to be only one signaling but to be multiple and complicated. EphA5 may mediate the below-mentioned various different pathways and may lead to affect both the downstream signaling which promotes ALP expression and that which suppresses simultaneously, although the overexpression of EphA5 suppressed ALP expression as a result. Alternative EphA or EphB signaling involved in osteogenic induction, and not just EphA5 signaling alone, may participate in this process. Given the variability of Eph expression in hBMSCs, the present results alone could not fully represent the specific mechanism of EphA5 signaling. More global approach is needed to define the exact role of EphA5 beyond this limitation.

Eph receptors are also known to signal through various different pathways and molecules, including small GTPases of the Rho and Ras family, focal adhesion kinase (FAK), the Jak/Stat pathway, and the PI3K pathway. In the present study, DEX treatment downregulated not only EphA5 but also downstream targets of EphA such as Rho/RAS-related genes (e.g., RASD1, ARHGAP, and RGNEF); this is significant findings. Activation of these proteins in various cell lines and carcinoma cells regulates cell-cell interactions through modulation of integrin activity and cell survival pathways [52–54], suggesting that they may perform a similar function in hBMSCs. Regulation of integrin activity has been demonstrated to be a key mechanism underpinning the effects of the ephrin and Eph system on cell-matrix adhesion and migration. For example, ephrin-A signaling activates the integrin pathway, thereby increasing cell adhesion or changing cell morphology and motility [55–57]. EphA-ephrin-A binding also induces integrin clustering for cell segregation during development [58]. Therefore, the EphA5 downregulation induced by DEX may be associated with ITGA5 upregulation [16] to increase osteoblast differentiation and osteogenesis in vitro. In the present study, EphA5 overexpression in hBMSCs decreased not only ITGA5 mRNA levels but also cell attachment to collagen I. This unclear mechanism for the adhesive effects should be fully explored; however, it might be partially involved in the ITGA5 level, which is supposed to be associated with cell adhesion and apoptosis according to the previous report [16]. We previously confirmed that population-selective effects of DEX enhanced the differentiation of BMSCs [14]. However, we did not characterize the selected cells or the mechanism of how they were selected in detail. Notably, we have also confirmed the osteogenic differentiation effects of DEX using immortalized human BMSCs, which are considered to be a single-cell-derived and homogenous population because they have been passaged numerous times (data not published). Studies using homogenous cell populations may depict a process different from our proposed mechanism of cell subpopulation selection by competitive proliferation.

As an alternative to subpopulation selection, we also observed that DEX augmented the responsiveness of hBMSCs to osteogenic stimulation by BMP in the previous study. In particular, DEX treatment promptly augmented BMP-induced phosphorylation of SMAD1/5/8 within 24 h but only scarcely affected the cell subpopulation distribution in the same time period [15]. DEX treatment also inhibited EphA5 mRNA expression by 6 hours, which was associated with increased levels of ALP in the present study. This rapid decrease in EphA5 levels suggests that DEX mediates a phenotypic switch from senescence to rapid growth in hBMSCs through targeting of this dormancy-associated marker EphA5, in addition to the previously observed subpopulation redistribution effect. The decrease in EphA5 levels may contribute to amelioration of cell senescence and to higher cell responsiveness to differentiation-inducing factors. Considering that not only repeating passages but also BMP-2 treatment induced EphA5 expression to some degree, this result seems to indicate that EphA5 is also a differentiation marker rather than a senescence-related marker. But first, there were times when BMP-2 treatment did not increase ALP expression on hBMSCs under certain conditions such as at low density (Figure 2(d)) or in some samples due to the individual responsiveness. Secondly, we have demonstrated that BMP-2 treatment could promote osteogenic differentiations of hBMSCs in a different manner, maintaining or promoting the overall expression profile of ephrins and Eph receptors whereas DEX treatment could have selective effects on ephrin/Eph subfamilies in hBMSCs. We speculate that BMP could upregulate other ephrin/Eph subfamilies or accelerate other ways of signaling which could have stronger effects than those of EphA5, leading to osteogenic differentiation despite increased EphA5 levels. Due to these strong and global effects of BMP-2 with an overall upregulation of many molecules, we think that EphA5 should not be regarded as a differentiation marker even if it could be increased by BMP-2. We observed that DEX treatment selectively inhibited not only mRNA expression of EphA5 but also EphA2, which was also known as an osteogenic inhibitor [7, 23], while DEX increased the levels of osteogenic stimulators such as ephrin-B2 [6] and EphA4 [8]. Apart from the effect of one of well-established osteogenic induction reagents, BMP-2, we have focused on the unclear and selective effect of DEX which could suppress osteogenic inhibitors in this paper. Further experiments must be needed to investigate the association between BMP signaling and ephrin/Eph subfamilies, and the mechanism of a synergetic effect of DEX and BMP-2.

Several studies have provided evidence that EphA5 is frequently downregulated in various cancer cell lines and tumor tissues via aberrant hypermethylation of its promoter [32, 34]. However, the nature of the stimulus that upregulates EphA5 over repeated passaging in BMSCs remains unclear. We indicated that histone deacetylation might be associated with the observed upregulation of EphA5. As one of limitations to our study, this VPA experiment is a global stimulus and the effect cannot be assigned to EphA5. Histone H3 acetylation at Lys9 and Lys14 (H3K9K14ac) or trimethylation at Lys9 and Lys27 (H3K9me3 and H3K27me3) in general correlates with open or closed chromatin state [59, 60]. We need to investigate posttranslational modification of histone H3 bound to the EphA5 promoters using chromatin immunoprecipitation (ChIP). Recently, miR-34a was reported to negatively modulate chondrogenesis by targeting EphA5 in chick limb mesenchymal cells [61], whereas antiangiogenic and dormancy-promoting molecules including EphA5 were reported to be upregulated by expression of dormancy-associated miR-580, miR-588, and miR-190 [37]. Further studies are thus required to determine the particular stimulus leading to upregulation of EphA5 over repeated passaging, including the potential role of miRNA.

In summary, in repeatedly passaged cultures, the upregulation of dormancy-associated EphA5 independent of methylation may inhibit signaling by other EphA or EphB family members through direct interactions, although competition for ligand binding may also occur. An imbalance between EphA5 and ligand expression may compromise Eph ligand-dependent differentiation processes (Figure 5) and may mediate ligand-independent processes in hBMSCs. Elucidation of the potential inhibitory function of EphA5 expressed in hBMSCs may provide an alternative approach for manipulating the fate of hBMSCs and for lineage differentiation in cell therapy strategies and regenerative medicine.

## Supplementary Material

S1 Figure: Forced expression of EphA5 does not affect EphA2 and EphA4 levels in hBMSCs.
S2 Figure: Treatment with various osteogenic induction reagents. Treatment with ascorbic acid and β-glycerophosphate affects ALP expression but not EphA5 expression. EphA5 expression was transiently reduced after 2 hours of BMP induction and gradually increased again. ALP expression was induced after 24 h of BMP induction.
S3 Figure: mRNA expression of integrin family members. ITGA5 was up-regulated during osteogenic induction (including DEX) of hBMSCs.

## Figures and Tables

**Table 1:** RT-PCR primers used in this study.

Genes	Forward	Reverse
*β*-act	TGGCACCCAGCACAATGAA	CTAAGTCATAGTCCGCCTAGAAGCA
ALP	GGACCATTCCCACGTCT	CCTTGTAGCCAGGCCCATTG
Runx2	CACTGGCGCTGCAACAAGA	CATTCCGGAGCTCAGCAGAATA
ephrin-A1	TGATCGCCACACCGTCTTC	CAGCGTCTGCCACAGAGTGA
ephrin-A2	CTGCCTGCGACTGAAGGTGTA	ACACGAGTTATTGCTGGTGAAGATG
ephrin-A3	TCTGAGGATGAAGGTGTTCGTCTG	TTCTCAAGCTTGGGCACCTG
ephrin-A4	TCGGCTTTGAGTTCTTACCTGGA	AGACACCTGGAGCCTCAAGCA
ephrin-A5	TGCTGGCATGTCGGAGGTTA	ACTGCAAAGCAGGGCAGTACAAG
ephrin-B1	CCAAGAACCTGGAGCCCGTA	AGATGATGTCCAGCTTGTCTCCAAT
ephrin-B2	CTGCTGGATCAACCAGGAATAAAGA	TCCTGAAGCAATCCCTGCAAATA
ephrin-B3	CTGTCTACTGGAACTCGGCGAATAA	CCGATCTGAGGGTACAGCACATAA
EphA1	CCTGTGCTGCAAGGTGTCTGA	GTGAAGATCCGATGGGCAATG
EphA2	GAGCTTTGGCATTGTCATGTGG	GCACTGCATCATGAGCTGGTAGA
EphA3	TTTGTCCTGGCAAGAACCTGAAC	TTCGGGCTCGGATTTGGA
EphA4	GCCGAGTGAGCTCCAATGCTA	GCCTGCATACACAAGGTGAAGCTA
EphA5	GCCCGGCAGTATGTGTCTGTAA	TCCATTGGGACGATCTGGTTC
EphA7	AGAACACTGTCCTCACACTTGACC	TGACAAGCATAAACCACCAGTTCTA
EphA8	CCTATGGAAGTCGGAAACATGGTC	AGAGCCCAGAAATTGGGTAAGAGTG
EphB1	GCCCAATGGCATCATCCTG	ATCAATCCTTGCTGTGTTGGTCTG
EphB2	GACCAAGAGCACACCTGTGATGA	CCACCAGCTGGATGACTGTGA
EphB3	AGACTCGGACTCTGCGGACA	GCTCACTCCACTCGAGGATCA
EphB4	ATGCCTGGAGTTACGGGATTG	TCCAGCATGAGCTGGTGGAG
EphB6	GACCAATGGGAACATCCTGGAC	CCCGCACCTGGAAACCATAG

**Table 2:** Genes upregulated in hBMSCs by DEX treatment. Human genes upregulated by at least threefold in hBMSCs treated with DEX for 6 h at P5 compared with nontreated cells.

Genes		Gene bank	Log_2_ ratio
FKBP5	FK506 binding protein 5	NM_004117	3.932
CYP19A1	Cytochrome P450, family 19, subfamily A, polypeptide 1, transcript variant 2	NM_031226	3.880
HEYL	Hairy/enhancer-of-split related to YRPW motif-like	NM_014571	3.162
BMP6	Bone morphogenetic protein 6	NM_001718	3.006
DKK1	Dickkopf homolog 1 (*Xenopus laevis*)	NM_012242	2.889
TNFAIP8L3	Tumor necrosis factor, alpha-induced protein 8-like 3	NM_207381	2.685
INHBB	Inhibin, beta B	NM_002193	2.653
MYPN	Myopalladin	NM_032578	2.594
CPM	Carboxypeptidase M, transcript variant 1	NM_001874	2.590
XIRP1	Xin actin-binding repeat containing 1	NM_194293	2.543
FOXO1	Forkhead box O1	NM_002015	2.413
CDH15	Cadherin 15, type 1, M-cadherin (myotubule)	NM_004933	2.377
GAL	Galanin prepropeptide	NM_015973	2.345
SNCAIP	Synuclein, alpha interacting protein	NM_005460	2.338
RNF128	Ring finger protein 128, transcript variant 1	NM_194463	2.293
PRICKLE2	Prickle homolog 2 (*Drosophila*)	NM_198859	2.289
ADRA1B	Adrenergic, alpha-1B-, receptor	NM_000679	2.267
PTGER2	Prostaglandin E receptor 2 (subtype EP2), 53 kDa	NM_000956	2.254
PPP1R14C	Protein phosphatase 1, regulatory (inhibitor) subunit 14C	NM_030949	2.234
WISP1	WNT1 inducible signaling pathway protein 1, transcript variant 2	NM_080838	2.220
ANGPTL4	Angiopoietin-like 4, transcript variant 1	NM_139314	2.201
DIRAS3	DIRAS family, GTP-binding RAS-like 3	NM_004675	2.173
ZNF469	Zinc finger protein 469	NM_001127464	2.145
CRYGS	Crystallin, gamma S	NM_017541	2.140
MT1M	Metallothionein 1M	NM_176870	2.121
SHC4	SHC (Src homology 2 domain containing) family, member 4	NM_203349	2.070
SGEF	Src homology 3 domain-containing guanine nucleotide exchange factor	NM_015595	2.046
TET3	Tet oncogene family member 3	NM_144993	2.019
PAG1	Phosphoprotein associated with glycosphingolipid microdomains 1	NM_018440	1.986
EEPD1	Endonuclease/exonuclease/phosphatase family domain containing 1	NM_030636	1.980
SLC20A1	Solute carrier family 20 (phosphate transporter), member 1	NM_005415	1.971
CMKLR1	Chemokine-like receptor 1, transcript variant 1	NM_001142343	1.970
FABP5	Fatty acid binding protein 5 (psoriasis-associated)	NM_001444	1.948
ABLIM3	Actin binding LIM protein family, member 3	NM_014945	1.944
SEC14L2	SEC14-like 2 (S. cerevisiae), transcript variant 1	NM_012429	1.942
CAMK2N1	Calcium/calmodulin-dependent protein kinase II inhibitor 1	NM_018584	1.906
HS3ST3B1	Heparan sulfate (glucosamine) 3-O-sulfotransferase 3B1	NM_006041	1.839
KBTBD11	Kelch repeat and BTB (POZ) domain containing 11	NM_014867	1.829
WNT5B	Wingless-type MMTV integration site family, member 5B, transcript variant 2	NM_030775	1.817
SLA	Src-like adaptor, transcript variant 1	NM_001045556	1.788
MT1G	Metallothionein 1G	NM_005950	1.781
MT1A	Metallothionein 1A	NM_005946	1.780
TOP1	Topoisomerase (DNA) I	NM_003286	1.771
MTSS1	Metastasis suppressor 1	NM_014751	1.765
BAIAP2	BAI1-associated protein 2, transcript variant 3	NM_006340	1.750
IRAK3	Interleukin-1 receptor-associated kinase 3, transcript variant 1	NM_007199	1.740
MT1B	Metallothionein 1B	NM_005947	1.734
FADS1	Fatty acid desaturase 1	NM_013402	1.725
ABTB2	Ankyrin repeat and BTB (POZ) domain containing 2	NM_145804	1.715
JARID2	Jumonji, AT rich interactive domain 2	NM_004973	1.686
ITGA5	Integrin, alpha 5 (fibronectin receptor, alpha polypeptide)	NM_002205	1.652
MT1X	Metallothionein 1X	NM_005952	1.647
GJB3	Gap junction protein, beta 3, 31 kDa, transcript variant 1	NM_024009	1.645
MT1H	Metallothionein 1H	NM_005951	1.639
HIVEP3	Human immunodeficiency virus type I enhancer binding protein 3	NM_024503	1.619
TCF7	Transcription factor 7 (T-cell specific, HMG-box), transcript variant 1	NM_003202	1.617
NR2F1	Nuclear receptor subfamily 2, group F, member 1	NM_005654	1.613

A total of 28,780 human genes consistent with the quality criteria, genes upregulated threefold or higher are listed.

**Table 3:** Genes downregulated in hBMSCs by DEX treatment. Human genes downregulated by at least threefold in hBMSCs treated with DEX for 6 h at P5 compared with nontreated cells.

Genes		Genebank	Log_2_ ratio
EGR2	Early growth response 2 (Krox-20 homolog, *Drosophila*), transcript variant 1	NM_000399	−4.446
CXCL1	Chemokine (C-X-C motif) ligand 1 (melanoma growth stimulating activity, alpha)	NM_001511	−4.060
EGR1	Early growth response 1	NM_001964	−3.989
CXCL2	Chemokine (C-X-C motif) ligand 2	NM_002089	−3.925
ATF3	Activating transcription factor 3, transcript variant 4	NM_001040619	−3.847
EGR3	Early growth response 3	NM_004430	−3.382
NR4A1	Nuclear receptor subfamily 4, group A, member 1, transcript variant 1	NM_002135	−3.312
IER3	Immediate early response 3	NM_003897	−3.294
SERTAD4	SERTA domain containing 4	NM_019605	−3.142
FOS	v-fos FBJ murine osteosarcoma viral oncogene homolog	NM_005252	−3.050
FOSB	FBJ murine osteosarcoma viral oncogene homolog B, transcript variant 1	NM_006732	−3.042
CXCL3	Chemokine (C-X-C motif) ligand 3	NM_002090	−2.950
ZC3H12A	Zinc finger CCCH-type containing 12A	NM_025079	−2.949
IL-8	Interleukin-8	NM_000584	−2.917
GDF15	Growth differentiation factor 15	NM_004864	−2.887
IL-6	Interleukin-6 (interferon, beta 2)	NM_000600	−2.886
MAP3K8	Mitogen-activated protein kinase kinase kinase 8	NM_005204	−2.837
BTG2	BTG family, member 2	NM_006763	−2.669
SEMA6D	Sema domain, transmembrane domain (TM), and cytoplasmic domain, (semaphorin) 6D, transcript variant 6	NM_024966	−2.659
PTGS2	Prostaglandin-endoperoxide synthase 2 (prostaglandin G/H synthase and cyclooxygenase)	NM_000963	−2.586
HES1	Hairy and enhancer of split 1, (*Drosophila*)	NM_005524	−2.520
HAS3	Hyaluronan synthase 3, transcript variant 2	NM_138612	−2.502
NCOA7	Nuclear receptor coactivator 7, transcript variant 1	NM_181782	−2.390
EYA1	Eyes absent homolog 1 (*Drosophila*), transcript variant 3	NM_000503	−2.376
JUNB	Jun B proto-oncogene	NM_002229	−2.356
C10orf10	Chromosome 10 open reading frame 10 (C10orf10)	NM_007021	−2.347
PIM1	Pim-1 oncogene	NM_002648	−2.316
ST8SIA1	ST8 alpha-N-acetyl-neuraminidase alpha-2,8-sialyltransferase 1	NM_003034	−2.256
C5orf41	Chromosome 5 open reading frame 41 (C5orf41)	NM_153607	−2.251
KLHL24	Kelch-like 24 (Drosophila)	NM_017644	−2.248
JUN	Jun oncogene	NM_002228	−2.247
BCL2L11	BCL2-like 11 (apoptosis facilitator), transcript variant 1	NM_138621	−2.227
EBF3	Early B-cell factor 3	NM_001005463	−2.195
NF*κ*BIZ	Nuclear factor of kappa light polypeptide gene enhancer in B-cells inhibitor, zeta, transcript variant 1	NM_031419	−2.188
AMOTL2	Angiomotin like 2	NM_016201	−2.160
TOX	Thymocyte selection-associated high mobility group box	NM_014729	−2.149
NUAK2	NUAK family, SNF1-like kinase, 2	NM_030952	−2.112
SASH1	SAM and SH3 domain containing 1	NM_015278	−2.112
SLC40A1	Solute carrier family 40 (iron-regulated transporter), member 1	NM_014585	−2.102
CCL2	Chemokine (C-C motif) ligand 2	NM_002982	−2.089
ID4	Inhibitor of DNA binding 4, dominant negative helix-loop-helix protein	NM_001546	−2.034
FAM110B	Family with sequence similarity 110, member B	NM_147189	−2.030
AXUD1	AXIN1 upregulated 1	NM_033027	−2.014
BCL3	B-cell CLL/lymphoma 3	NM_005178	−2.011
ARL4C	ADP-ribosylation factor-like 4C	NM_005737	−2.011
IER2	Immediate early response 2	NM_004907	−2.009
RASD1	RAS, dexamethasone-induced 1	NM_016084	−2.007
OSR2	Odd-skipped related 2 (*Drosophila*), transcript variant 2	NM_053001	−1.985
GAB1	GRB2-associated binding protein 1, transcript variant 1	NM_207123	−1.977
TNFAIP3	Tumor necrosis factor, alpha-induced protein 3	NM_006290	−1.954
PDK4	Pyruvate dehydrogenase kinase, isozyme 4	NM_002612	−1.930
BMP4	Bone morphogenetic protein 4, transcript variant 1	NM_001202	−1.867
BDKRB1	Bradykinin receptor B1	NM_000710	−1.849
IRF1	Interferon regulatory factor 1	NM_002198	−1.840
N4BP2L1	NEDD4 binding protein 2-like 1, transcript variant 1	NM_052818	−1.814
ARHGAP20	Rho GTPase activating protein 20	NM_020809	−1.802
NF*κ*BIA	Nuclear factor of kappa light polypeptide gene enhancer in B-cells inhibitor, alpha	NM_020529	−1.801
CXCR7	Chemokine (C-X-C motif) receptor 7	NM_020311	−1.789
RFTN2	Raftlin family member 2	NM_144629	−1.774
PRICKLE1	Prickle homolog 1 (*Drosophila*)	NM_153026	−1.768
TIAM2	T-cell lymphoma invasion and metastasis 2, transcript variant 1	NM_012454	−1.753
BIRC3	Baculoviral IAP repeat-containing 3, transcript variant 1	NM_001165	−1.751
C5orf4	Chromosome 5 open reading frame 4, transcript variant 2	NM_032385	−1.731
GATA6	GATA binding protein 6	NM_005257	−1.729
CLDN23	Claudin 23	NM_194284	−1.719
TNFRSF11B	Tumor necrosis factor receptor superfamily, member 11b	NM_002546	−1.700
C2orf67	*Homo sapiens* chromosome 2 open reading frame 67 (C2orf67), mRNA [NM_152519]	NM_152519	−1.693
TRIB3	Tribbles homolog 3 (*Drosophila*)	NM_021158	−1.693
EBF1	Early B-cell factor 1	NM_024007	−1.684
PRAGMIN	Homolog of rat pragma of Rnd2	NM_001080826	−1.683
MAFB	v-maf musculoaponeurotic fibrosarcoma oncogene homolog B (avian)	NM_005461	−1.680
ZFP36	Zinc finger protein 36, C3H type, homolog (mouse)	NM_003407	−1.673
IL-7	Interleukin-7	NM_000880	−1.642
RUNX1T1	Runt-related transcription factor 1; translocated to, 1 (cyclin D-related), transcript variant 1	NM_004349	−1.628
HK2	Hexokinase 2	NM_000189	−1.623

EPHA5	EPH receptor A5, transcript variant 1	NM_004439	−1.186

A total of 28,780 human genes consistent with the quality criteria, genes downregulated twofold or higher are listed.
