# Supplementary material for: Dexamethasone Regulates EphA5, a Potential Inhibitory Factor with Osteogenic Capability of Human Bone Marrow Stromal Cells
Source: Stem Cells Int. 2016 Jan 10;2016:1301608. doi: 10.1155/2016/1301608 (PMC4736961; doi:10.1155/2016/1301608)

**Supplementary Materials**

S1 Figure. Forced expression of EphA5 does not affect EphA2 and EphA4 levels in hBMSCs.

(A, B) Quantitative analysis of mRNA expression of osteogenic markers at 7 days of osteogenic culture: (A) EphA2, and (B) EphA4 (*n*=4).

The fold change of gene expression was normalized against the expression in cell cultures without LV transduction.

S2 Figure. Treatment with various osteogenic induction reagents.

(A, B) Quantitative analyses of the mRNA expression of (A) ALP and (B) EphA5.

Osteogenic medium contained 10 mM β-glycerophosphate and 50 µg/ml ascorbic acid phosphate. Treatment with ascorbic acid and β-glycerophosphate affects ALP expression but not EphA5 expression.

The fold change of gene expression was normalized against the expression in the control P1 cell culture at day 0 (*n*=5).

(C) Quantitative analyses of ALP and EphA5 mRNA expression in BMSCs at passage 5 when treated with BMP-2.

The change in the expression of each gene expression was normalized against the expression in cell cultures prior to BMP addition (*n*=4).

EphA5 expression was transiently reduced after 2 hours of BMP induction and gradually increased again. ALP expression was induced after 24 h of BMP induction.

S3 Figure. Expression of integrin family members.

1. Quantitative analysis of the mRNA expression of integrin family members.

Osteogenic medium contained 10 mM β-glycerophosphate, 50 µg/ml ascorbic acid phosphate, and 100 nM DEX.

The fold change of gene expression was normalized against the expression in the control P1 cell culture at day 0 (*n*=6).

ITGA5 was up-regulated during osteogenic induction of hBMSCs.


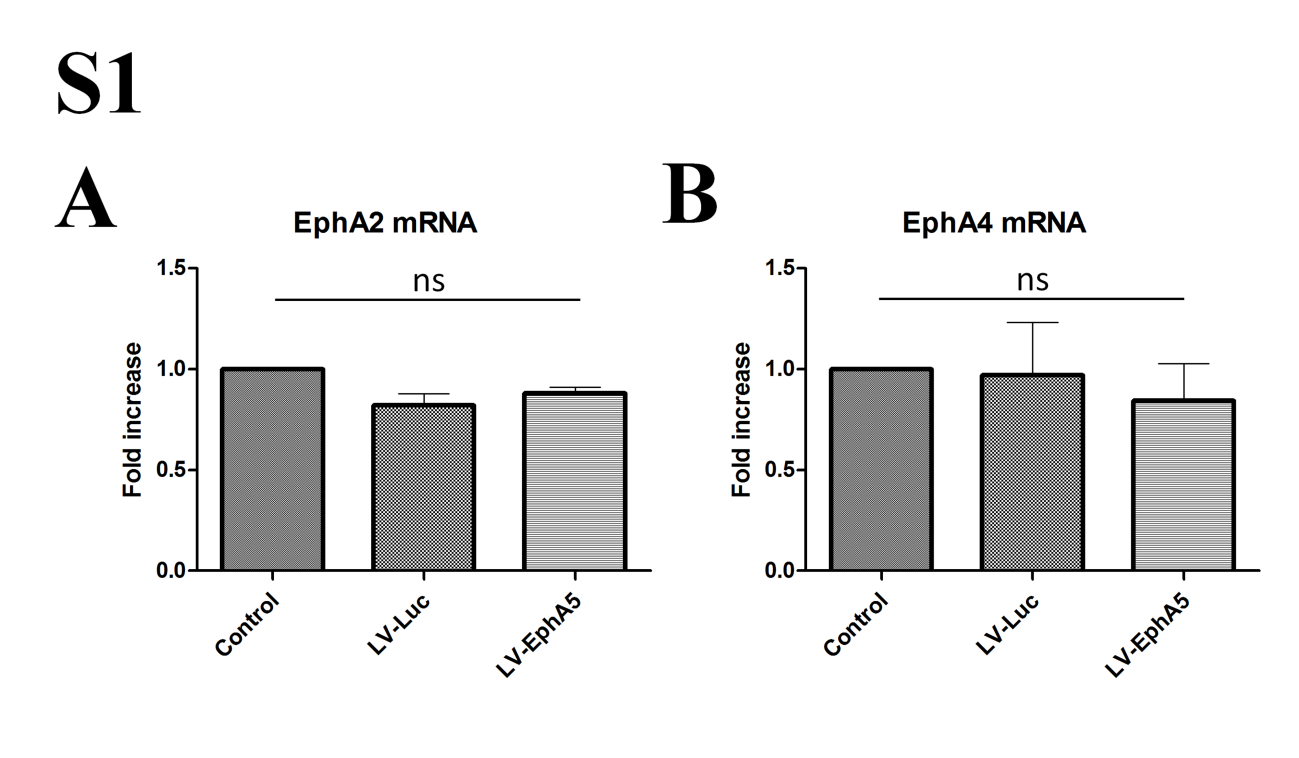


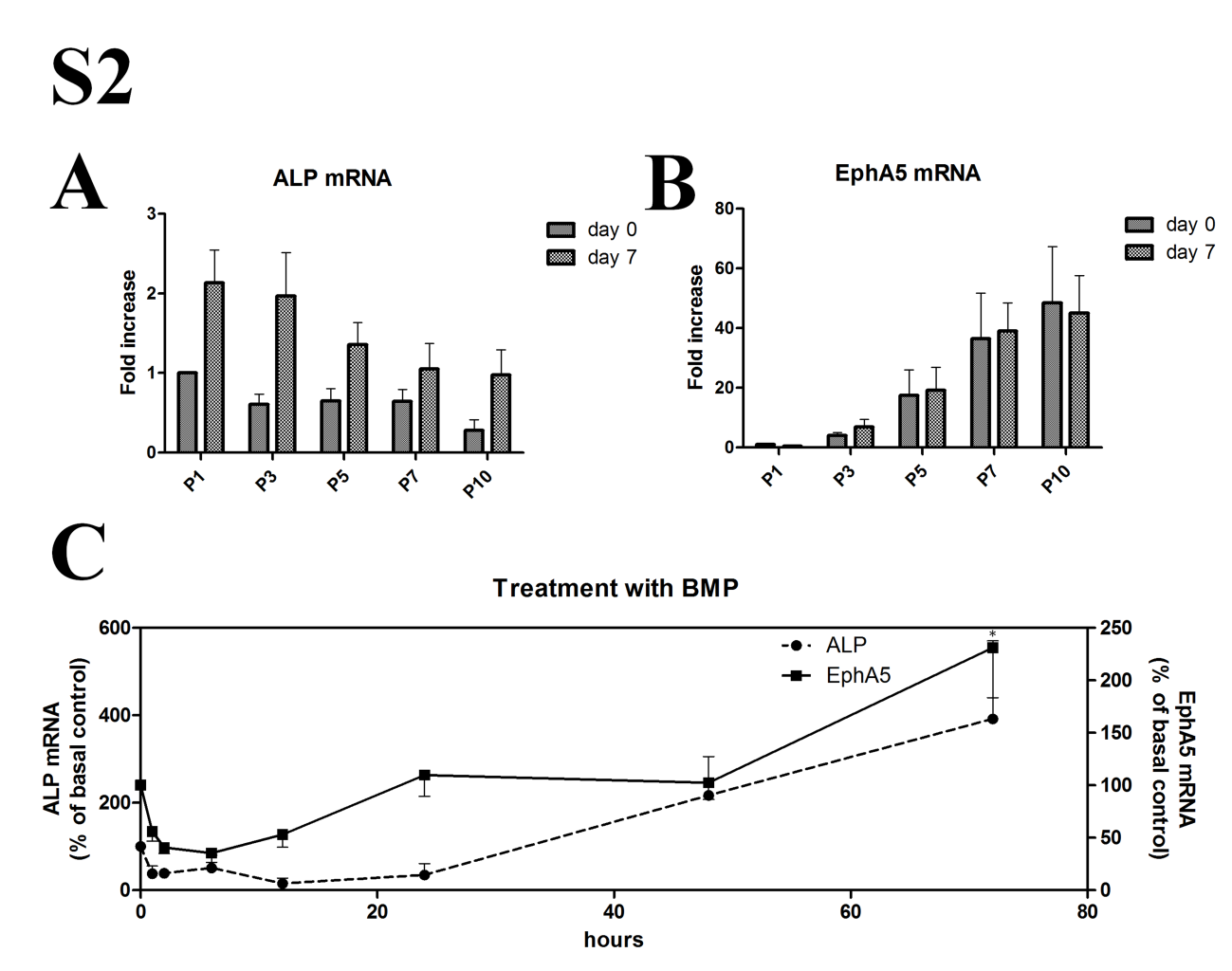


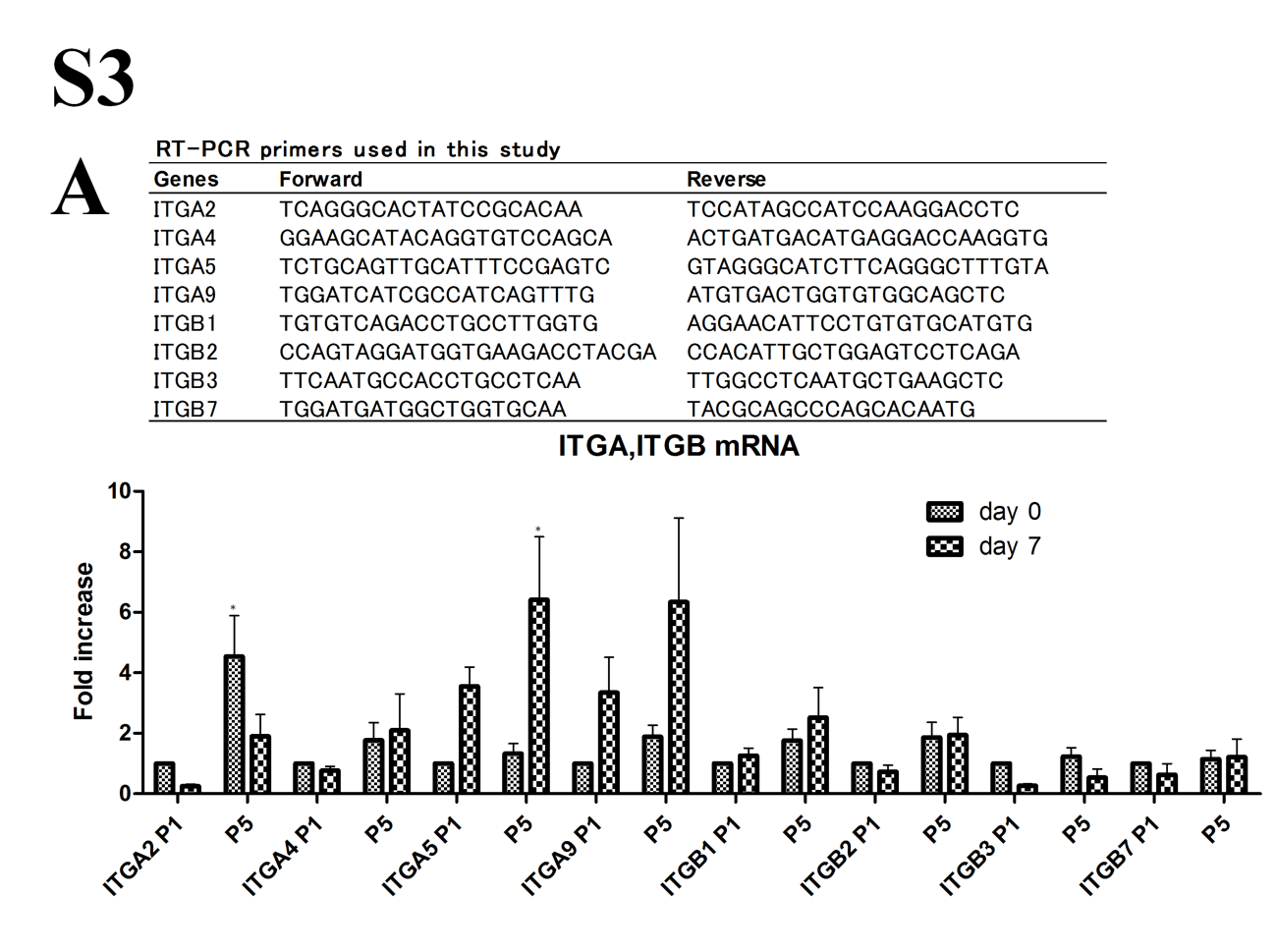

Supplement: Supplementary file 1 — S1 Figure: Forced expression of EphA5 does not affect EphA2 and EphA4 levels in hBMSCs. S2 Figure: Treatment with various osteogenic induction reagents. Treatment with ascorbic acid and β-glycerophosphate affects ALP expression but not EphA5 expression. EphA5 expression was transiently reduced after 2 hours of BMP induction and gradually increased again. ALP expression was induced after 24 h of BMP induction. S3 Figure: mRNA expression of integrin family members. ITGA5 was up-regulated during osteogenic induction (including DEX) of hBMSCs. [file 1301608.f1.docx]
